# COlchicine to Prevent PeriprocEdural Myocardial Injury in Percutaneous Coronary Intervention (COPE-PCI): Coronary Microvascular Physiology Pilot Substudy

**DOI:** 10.1155/2022/1098429

**Published:** 2022-05-29

**Authors:** Justin Cole, Nay Htun, Robert Lew, Mark Freilich, Stephen Quinn, Jamie Layland

**Affiliations:** ^1^Peninsula Heart Service, Peninsula Health, Frankston, Australia; ^2^Peninsula Clinical School, Monash University, Melbourne, Australia; ^3^Department of Health Science and Biostatistics, Swinburne University of Technology, Melbourne, Australia

## Abstract

**Aim:**

In this randomized pilot trial, we aimed to assess the anti-inflammatory effect of preprocedural colchicine on coronary microvascular physiology measurements before and after PCI.

**Methods:**

Patients undergoing PCI for stable angina (SA) or non-ST-elevation myocardial infarction (NSTEMI) were randomized to oral colchicine or placebo, 6- to 24-hours before the procedure. Strict prespecified inclusion/exclusion criteria were set to ensure all patients were given the study medication, had a PCI, and had pre- and post-PCI culprit vessel invasive coronary physiology measurements. Fractional flow reserve (FFR), Index of Microvascular Resistance (IMR), Coronary Flow Reserve (CFR), and Resistive Reserve Ratio (RRR) were measured immediately before and after PCI. CMVD was defined as any one of post-PCI IMR >32 or CFR <2 or RRR <2. High-sensitive-(hs)-troponin-I, hsCRP, and leucocyte count were measured before and 24 hours after PCI.

**Results:**

A total of 50 patients were randomized and met the strict prespecified inclusion/exclusion criteria: 24-colchicine and 26-placebo. Pre-PCI coronary physiology measurements, hs-troponin-I, and hsCRP were similar between groups. Although numerically lower in patients given colchicine, the proportion of patients who developed CMVD was not significantly different between groups (colchicine: 10 (42%) vs placebo: 16 (62%), *p*=0.16). Colchicine patients had higher post-PCI CFR and RRR vs placebo (respectively: 3.25 vs 2.00, *p*=0.03 & 4.25 vs 2.75, *p* < 0.01). Neutrophil count was lower after PCI in the colchicine arm (*p*=0.02), and hsCRP post-PCI remained low in both treatment arms (1.0 mg/L vs 1.7 mg/L, *p*=0.97). Patients randomized to colchicine had significantly less PCI-related absolute hs-troponin-I change (46 ng/L vs 152 ng/L, *p*=0.01).

**Conclusion:**

In this pilot randomized substudy, colchicine given 6 to 24 hours before PCI did not statistically impact the post-PCI CMVD definition used in this study, yet it did improve post-PCI RRR and CFR measurements, with less procedure-related troponin release and less inflammation.

## 1. Introduction/Background

Percutaneous coronary intervention (PCI), of a functionally significant epicardial artery stenosis, is one of the main treatments for symptomatic patients with either stable coronary disease or acute coronary syndromes (ACSs) [1, 2]. However, PCI can activate multiple pathways leading to coronary microvascular dysfunction (CMVD) [3] with associated adverse clinical outcomes [3, 4].

CMVD is defined by impaired response of coronary microvascular flow to vasodilator stimuli [5] which can occur through many pathways as a consequence of PCI [3, 5] and is associated with myocardial injury [4, 6, 7], yet the underlying mechanisms of this association remain under investigation. Small trials offering targeted treatment aimed at reducing PCI-related CMVD have been performed and have shown promise. Both ACE inhibitors and statins have demonstrated improved measures of microvascular function in patients undergoing PCI [8–12], and a strategy of direct stenting has also been shown to have favorable effects on PCI-related coronary microvascular dysfunction, as measured by IMR [13].

In the COPE-PCI Pilot Trial [14], we have previously demonstrated that when colchicine is given before PCI, it is associated with reductions in periprocedural myocardial injury and in a subsequent study is associated with numerically lower pre-PCI levels of inflammation [15]. Moreover, we have previously demonstrated a relationship between pre-PCI microvascular function and inflammation [16]. Thus, in this substudy of the COPE-PCI Pilot Trial, we aimed to assess the anti-inflammatory effect of colchicine, given 6–24 hours before PCI, on coronary microvascular physiology measurements before and after PCI. We hypothesized that colchicines' anti-inflammatory effect would attenuate PCI-associated impairment in coronary microvascular function.

## 2. Methods

### 2.1. Study Population

The study population consisted of both stable angina (SA) patients intended for elective PCI and patients presenting with non-ST-elevation myocardial infarction (NSTEMI) planned for in-patient coronary angiography and PCI. Patients were assessed against strict inclusion and exclusion criteria (see below) and must have had a lesion appropriate for invasive coronary physiological measurements, deemed by the interventional cardiologist at the time of the index PCI. Notable inclusion criteria were patients with a de novo lesion amenable to PCI, and patients with high-sensitive (hs) troponin-I and creatinine kinase (CK) that had peaked and stabilized prior to PCI. Patients were excluded if they had left main disease and required bifurcation lesion PCI, or the culprit's vessel was completely occluded. Additionally, patients were excluded if they had heavily calcified or tortuous vessels such that safe passage of the pressure wire could not be guaranteed. Furthermore, patients were excluded if they had active inflammation or infection or were taking anti-inflammatory medications, had prior ACS within 12-months, had severe renal impairment (creatinine clearance <45 ml/min), and developed ST-elevation prior to PCI or troponin increase after randomization and prior to PCI. Patients received dual antiplatelet therapy prior to PCI. We prespecified that patients who were initially enrolled in the trial but who did not go on to PCI or invasive coronary physiological measurements were excluded from statistical analysis.

### 2.2. Study Protocol

All patients reviewed and signed an informed consent prior to randomization and PCI.

Consenting patients were randomized 1 : 1 to oral colchicine (1 mg followed by 0.5 mg one hour later [14]) or placebo, 6 to 24 hours prior to a coronary angiogram. Sequential randomization was performed using proprietary software (https://www.sealedenvelope.com). Study patients were assigned to colchicine or placebo prior to coronary angiography by an independent research assistant not involved in the invasive protocol. Randomization was simple and not stratified. Study medication was denoted Drug A or Drug B throughout the trial. Both Drug A and Drug B were in labeled bottles and looked identical. This was a double-blind randomized placebo-controlled trial. All parties involved were blinded to the treatment allocation until the trial had finished. The study protocol was approved by the Human Research Ethics Committee at St Vincent's Hospital Melbourne and Frankston Hospital Melbourne, Australia. The trial was publicly registered (COPE-PCI Trial ANZCTR Trial ID: ACTRN12615000485538). The trial ran from 1 December 2017 to 30 December 2019.

### 2.3. PCI Procedure

All patients received weight-adjusted bolus (100 Units/kg) intravenous heparin, immediately prior to PCI, and additional bolus dosing to maintain an activated clotting time of >250 secs. Technical aspects of the PCI procedure were determined by the practicing interventionalist.

### 2.4. Invasive Coronary Physiological Measurements [3, 17–19]

We performed invasive interrogation of the culprit vessel coronary circulation as previously described [20]. Measurements were taken both before and after PCI. In brief, a dual temperature and pressure-sensing guidewire was used to cross the lesion, measuring distal pressure (Pd) and transit time (Tmn) of 3 ml of room temperature heparinized saline-injected intracoronary. Three reproducible and consistent thermodilution curves were performed before and after PCI, both at rest (_Rest_) and at maximal hyperemia (_Hyp_). Maximal hyperemia was induced by the administration of intravenous adenosine at 140 ug/kg/min for up to 2 minutes and confirmed by clinical response and hemodynamic changes. Proximal pressure was obtained from the guiding catheter (Pa). Coronary artery wedge pressure (Pw) was obtained during a 20-second balloon occlusion of the culprit's vessel during the initial balloon inflation.

(i) Fractional Flow Reserve myocardial (FFRmyo) [17] was measured at maximal hyperemia, defined as follows: FFRmyo = Pd_Hyp_/Pa_Hyp._
(ii) Coronary Flow Reserve (CFR_thermo_) was defined as CFR_thermo_ = Tmn_Rest_/Tmn_Hyp_ [19]
(iii) In the presence of severe epicardial stenosis, the index of microvascular resistance (IMR) was defined by incorporating Pw via the following equation:IMR = Pa_Hyp_ × Tmn_Hyp_ × FFRcor [18]. FFR coronary (FFRcor) was defined by the following equation: FFRcor = (Pd_Hyp_-Pw)/(Pa_Hyp_ − Pw) [18], where Pw was not recorded and FFRcor was predicted by FFRmyo using the following equation [17]:FFRcor = (1.34 × FFRmyo) − 0.32. Therefore, in the absence of Pw: IMR = Pa_Hyp_ × Tmn_Hyp_ × ((1.34 × FFRmyo) − 0.32).
(iv) Baseline Resistance Index (BRI) [20] was defined as per IMR; however, baseline measures were used:
  - BRI = Pa_Rest_ × Tmn_Rest_ × ((Pd_Rest_ − Pw)/(Pa_Rest_ − Pw)).
  - Or in the absence of Pw: BRI = Pa_Rest_ × Tmn_Rest_ × (((1.34 × (Pd_Rest_/Pa_Rest_)) − 0.32)
(v) Resistive Reserve Ratio (RRR) [20] was defined by the following equation: RRR = BRI/IMR.

### 2.5. Blood Sampling

All patients had study blood samples collected immediately prior to PCI (before administration of PCI procedure-related medications) and 24 hours later. All patients also had standard routine clinical blood tests. Study blood samples were assessed for high sensitivity (hs)-troponin-I (ng/L), hsCRP (mg/L), and leucocyte count. Serum hsCRP level was quantified using a Human CRP Simplex ProcartaPlex™ immunoassay that utilizes Luminex™ xMAP technology for protein detection/quantitation. We prespecified that patients enrolled in the trial who had incomplete blood sampling were excluded from statistical analysis.

### 2.6. Study Endpoints

The primary outcome of this study was the difference in proportion between treatment arms, of patients with coronary microvascular dysfunction, defined as any one of the following: post-PCI RRR <2.0, CFR <2.0, or IMR >32.

Additionally, colchicines' effect on coronary microvascular function was assessed by comparing pre- and post-PCI invasive coronary physiological measurements (listed above) between the treatment arms of this study.

Demographic, clinical, medication use, and PCI procedure data were recorded.

Inflammation was assessed by measuring pre-PCI and 24-hour post-PCI hsCRP and leucocyte count, comparing treatment arms.

Myocardial injury was assessed by measuring pre-PCI and 24-hour post-PCI hs-troponin-I and calculating absolute hs-troponin-I change: calculated as post-PCI measurements minus pre-PCI measurements, in each treatment arm of this study.

Patients were followed up for 24 hours after the procedure. All patients received standard medical and PCI therapy and were reviewed by their referring cardiologist within 30 days after PCI.

### 2.7. Statistical Analysis

Continuous variables are reported as mean ± SD or median (LQ-UQ) according to whether the variable is normal or skewed, and categorical variables are as numbers (*n* (%)). Differences in continuous variables were tested via an independent-samples *t*-test (normal data) or Mann–Whitney *U* test (skewed data). Similarly, paired variables were tested via a paired-samples *t*-test (normal data) or Wilcoxon signed-rank test (skewed data). Proportional differences in categorical variables were compared using the Chi-Square test or the Fisher's-Exact Test (Fisher's Exact Test was used when one or more cell frequencies were less than five). Assessment of bivariate correlation was performed using a Pearson Correlation Coefficient for normal data or a Spearman Correlation Coefficient for skewed data. A *p* value <0.05 (two-sided) was considered statistically significant. Statistical analyses were performed with IBM SPSS Statistics for Windows, Version 25.0 (Armonk, NY: IBM Corp).

## 3. Results

### 3.1. Study Population

**Figure 1:**
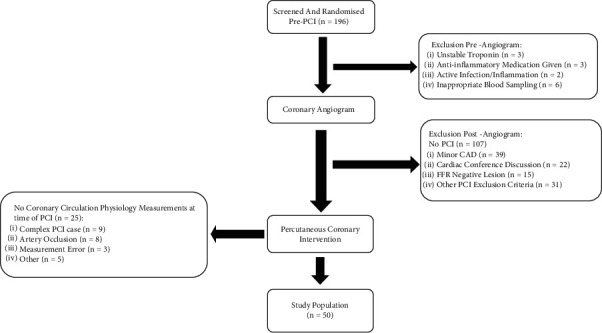
Overview of study design and patient allocation. (i) Development of unstable troponin or active inflammation/infection, anti-inflammatory medications given, or inappropriate blood sampling occurred in 14 patients after randomization. (ii) Cardiac conference discussion = patient had severe CAD at coronary angiography. The procedure was therefore stopped, and the patient's case was discussed at a later date at a combined cardiology/cardiothoracic surgical meeting to determine an optimal revascularization strategy. Most patients were sent for coronary artery bypass grafting. (iii) Other PCI exclusion criteria = see “exclusion criteria” in the supplemental material. (iv) Complex PCI case = as deemed by the interventional cardiologist, not appropriate for coronary artery physiology measurements.

A total of 196 patients were screened. Of these, 14 patients (7%) developed clinical criteria excluding them, 107 (54%) did not have PCI, and 25 (13%) did not have invasive coronary physiological measurements (see Figure 1). Thus, the final population was 50 patients, thereby ensuring all analyzed patients fulfilled the prespecified criteria: (1) received the study medication, (2) PCI procedure performed, and (3) complete pre- and post-PCI invasive coronary physiological measurements performed. Twenty-four (11 (46) NSTEMI, 13 (54) SA) were randomized to colchicine, 26 (12 (46) to NSTEMI, and 14 (54) SA) to placebo (Table 1).

Patients were well matched, without significant differences, for baseline characteristics and pre-PCI medication use (Table 1). There was no significant difference in PCI characteristics between treatment arms (Table 2).

Prior to PCI, there was no statistical difference in the invasive coronary physiological measurements between treatment groups (Table 3). FFR was similar and functionally significant in both treatment arms before PCI (0.65 vs 0.72, *p*=0.42), and IMR measures were low (<22).

Post-PCI patients randomized to colchicine had higher CFR and RRR compared to placebo (respectively: 3.25 (2.08–4.40) vs 2.00 (1.48–3.30), *p*=0.03 and 4.25 (2.45–5.24) vs 2.75 (1.67–3.44), *p* < 0.01, Table 3). Median IMR remained low in both treatment arms (IMR <20).

Comparing change between pre- and post-PCI invasive coronary physiological measurements, patients randomized to colchicine had significantly less change in Tmn_Rest_ measurements (0.01 vs −0.31, *p*=0.03) and significantly greater change in CFR (1.20 (−0.30–2.10) vs 0.10 (−0.70–0.63), *p*=0.03) (Table 3). There was a trend in favor of a change in RRR being numerically higher in the colchicine arm (*p*=0.054, Table 3).

Following PCI, there was no statistical difference in the proportion of patients with CMVD between those randomized to colchicine or placebo (colchicine: 10 (42%) vs placebo: 16 (62%), *p*=0.16, Table 4). This was also true for the proportion of patients with individual measures of CFR <2.0, IMR >32, or RRR <2.0 (Table 4).

Troponin measures prior to PCI were similar and low in both treatment groups (<13, Table 3). Most patients had a troponin elevation of any magnitude after PCI (colchicine: 75% vs placebo 92%, *p*=0.13). Patients randomized to colchicine had significantly less absolute troponin change after PCI (46 (1–154) vs 152 (48–633), *p*=0.01), compared to placebo (Table 3). No correlation was found between measurements of post-PCI troponin or absolute change in troponin with any invasive coronary physiological measurements comparing treatment groups. A weak negative correlation was found between absolute troponin change and change in Tmn_Rest_, in the overall cohort: Spearman's Correlation Coefficient: −0.31, *p*=0.03.

Patients randomized to colchicine had significantly lower pre-PCI total white blood cell (WBC) count: 6.7 ± 1.3 vs 7.7 ± 1.5, *p*=0.02 (Table 3). However, prior to PCI, the degree of inflammation was low and similar between treatment arms (median hsCRP = 1.1 mg/L). Neutrophil count was lower after PCI in the colchicine arm (*p*=0.02). Total WBC count and hsCRP were similar between treatment groups after PCI (Table 3), and hsCRP remained low.

**Figure 2:**
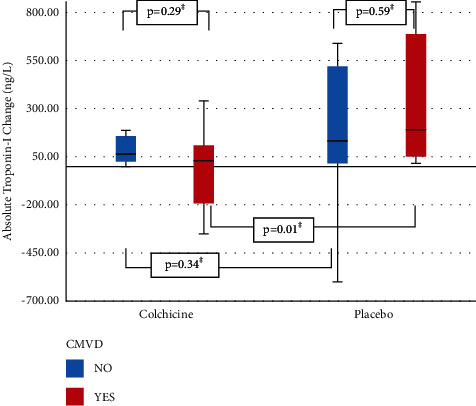
Boxplot of absolute hs-Troponin-I change in patients with or without post-PCI CMVD, according to study drug randomization.

Comparing patients who developed after PCI CMVD to those without CMVD, regardless of study drug treatment, there was no statistical difference in absolute troponin change (*p*=0.66), pre- or post-PCI neutrophil count (*p*=0.06 or 0.42), pre- or post-PCI WBC count (*p*=0.23 or 0.99), or pre- or post-PCI hsCRP (*p*=0.95 or 0.91). However, in the subset of patients who develop CMVD after PCI, absolute troponin change was less if they were given colchicine vs placebo (29 ng/L (−197-108) vs 189 (52–687), *p*=0.01, Figure 2).

### 3.2. Safety and Adverse Events

No adverse events were reported in any study participants.

## 4. Discussion

In this study, we found several pertinent and important findings. Firstly, preprocedural administration of colchicine had no impact on the development of PCI-induced coronary microvascular dysfunction (as defined in our study by any one of the following: post-PCI RRR <2.0, CFR <2.0, or IMR >32), yet colchicine was found to improve post-PCI CFR and RRR measures. Additionally, patients randomized to colchicine had less procedurally related absolute troponin change and lower neutrophil count. Furthermore, of the subset of patients who had CMVD after the procedure, those given colchicine had significantly lower absolute troponin change after PCI.

Although colchicine failed to reach its primary endpoint by modifying PCI-related CMVD in this pilot substudy, this may be explained by the fact that this patient group was lower in risk, as reflected by low levels of IMR before and after PCI, and so the true effect size of colchicine may have been attenuated. However, an interesting observation in our study was that preprocedural colchicine seemed to improve post-PCI CFR and RRR, had no effect on FFR or IMR, and had a muted effect on change in Tmn_Rest_ compared to the placebo group whereas FFR assesses the functional significance of epicardial stenosis [17], and CFR and to a lesser extent RRR are influenced by both functional changes in epicardial and microcirculation compartments, yet CFR is not microvascular specific [21]. On the other hand, IMR assesses coronary microvascular resistance independent of epicardial arterial stenosis [18], under only hyperaemic conditions. Resistive Reserve Ratio, a newer marker of coronary physiology, represents the dynamic vasodilatory capacity [20] of the coronary microcirculation and the cumulative functional disease burden throughout the interrogated vessel [22]. Additionally, in this study, change in Tmn_Rest_ was reduced by a greater magnitude in the placebo arm compared to the colchicine arm. This may represent a nondynamic static increase in resting coronary vascular flow after PCI in the placebo group who had increased procedural-related myocardial injury, compared to the colchicine group who appear to retain a dynamic functional coronary vasculature. Thus, this study alludes to colchicine's improved functional effect on vascular dynamics (RRR), rather than influencing microvascular resistance at maximal hyperemia (IMR).

In the long term, therapies that improve RRR may have prognostic benefits, highlighted by recent studies showing the superior ability of RRR to have a prognostic role in both stable [22] and unstable [6, 23] coronary artery diseases, particularly in STEMI patients [20].

Results of published therapies aimed at attenuating measures of coronary microvascular function have been mixed, most have not assessed RRR, and as yet specific targeted anti-inflammatory medications have not been trialed. A recent meta-analysis found overall long-term use of calcium channel blocking medications improves CFR [24], and results with beta-blocker medications are mixed [24–26]. ACE inhibitors are effective at improving CFR [8, 9, 24] and IMR [9] alone. Statin therapy has been shown to (1) improve CFR with reduced inflammatory cytokines [10] and (2) improve IMR with reduced post-PCI troponin [11]. However, other statin studies have not been as convincing. A recent trial of women, without obstructive coronary disease, demonstrated no change in IMR and only an improvement in CRP after pretreatment with rosuvastatin [12]. Furthermore, a strategy of direct stenting has been shown to attenuate PCI-related CMVD [13] without an associated impact on troponin release. Additionally, Ticagrelor given for 6 months after PCI among patients with ACS improved CFR and IMR [27] compared to clopidogrel. Also, in patients presenting with STEMI, both intracoronary nicorandil and streptokinase have been shown to improve IMR [28, 29]. While these trialed therapies are not inflammation specific, there are many inflammatory mechanisms activated as a consequence of PCI that disturb microvascular function, such as endothelial dysfunction, increased oxidative stress, embolization of immunogenic debris, and induction of distal and systemic inflammation [3, 5]. Moreover, we have previously demonstrated a positive correlation between pre-PCI microvascular function (as measured by IMR) and inflammation [16]. Thus, strategies aimed at targeting inflammation before PCI may also attenuate PCI-related CMVD.

In our study, involving both stable patients and those with NSTEMI, colchicine appeared to aid in the restoration of PCI-related microvascular reactivity and coronary vascular flow reserve, seen as improved RRR and CFR. Colchicine's effect on the arteriolar function is supported by studies showing improvement of arterial wall stiffness in patients with Familial Mediterranean Fever, given higher doses of colchicine [30], and improvement of Flow-Mediated Vasodilation in patients with coronary artery disease and a higher degree of inflammation defined by a high white cell count [31]. These findings of improved vascular tone and improvements in endothelial function in colchicine-treated patients may partly explain the improvements seen in our study, namely, higher CFR and RRR after PCI among patients treated with preprocedural colchicine.

Not only does colchicine exert its effects through the inhibition of the NLRP3 inflammasome, but colchicine also suppresses neutrophil extracellular traps (NETs) formation in patients presenting with ACS treated with PCI [32]. This novel anti-inflammatory effect of colchicine may also assist in improving periprocedural coronary microvascular function and reducing periprocedural myocardial injury.

In these COPE-PCI trials, we have shown that when colchicine is given before PCI, it is associated with reductions in periprocedural myocardial injury [14] and numerically lower pre-PCI levels of inflammation [15]. In this final study, we show targeting inflammation with preprocedural colchicine, which is associated with improved coronary microvascular function as measured by RRR and CFR; however, it did not attenuate PCI-related CMVD.

## 5. Limitations

In this pilot study, the patient population consisted of a low-risk cohort for coronary microvascular dysfunction, with a combined high use of calcium channel and/or beta-blocker medications in the 24 hours prior to PCI. Additionally, we cannot comment on preprocedural drug posology. Pre-PCI IMR measures were low indicating that patients were at low risk of myocardial injury [7] and inflammation [16]. Also, notably, this study population had low levels of inflammation after the procedure, denoted by low hsCRP levels. Therefore, in our study population, the possible effect size of colchicine on coronary microvascular function may have been attenuated.

Due to the small population size in this pilot study, we had a lower number of patients with abnormal coronary microvascular function. It is possible that we would have observed a different result where there had been a larger number of patients with abnormal microvasculature.

A number of patients were randomized to study medication who ultimately were excluded due to prespecified inclusion/exclusion criteria, procedural necessities, and time frames. This was unavoidable and expected. In this pilot study, the coronary anatomy was not known prior to randomization and many patients evidently did not require PCI or have a suitable lesion to perform invasive coronary physiology measurements. This was anticipated prior to the commencement of this study, and it was prespecified that the final trial analysis would not include these patients, a methodology that has previously been published by other authors assessing PCI-induced periprocedural myocardial injury [33]. The reported results should therefore be interpreted with this in mind.

## 6. Conclusion

In this pilot substudy, administration of preprocedural colchicine improved post-PCI RRR and CFR, with less periprocedural absolute troponin change and lowered neutrophil count, without effect on the development of post-PCI Coronary Microvascular Dysfunction (defined in this study as any one of post-PCI IMR >32 or CFR <2 or RRR <2).

### 6.1. Impact on Daily Practice

Treatment of inflammation and impaired coronary microvascular function at the time of percutaneous coronary intervention (PCI) could improve outcomes. Based on this small, randomized placebo-controlled trial, improvements in post-PCI coronary microvascular function (Resistive Reserve Ratio (RRR) and Coronary Flow Reserve (CFR)), inflammation, and troponin release were seen in patients treated with preprocedural colchicine compared to placebo. Importantly, an improved RRR represents an improved vasodilatory capacity of the coronary microcirculation and improved functional disease burden throughout the interrogated vessel. Improved RRR in other studies has shown prognostic utility.

## 7. Inclusion and Exclusion Criteria

### 7.1. Inclusion Criteria

(i) Male or female over 18 years of age
(ii) Stable angina (SA) patients: symptomatic patients with stable angina or asymptomatic patients with positive functional tests (requiring elective PCI)
(iii) Non-ST-elevation myocardial infarction (NSTEMI) patients: defined as the recent onset of chest pain associated with ST-segment and/or T-wave ECG changes and positive cardiac enzymes (high sensitivity troponin)
  - Troponin must have peaked and stabilized prior to PCI, as defined by two serial Troponin measures
  - Stable troponins are defined by ≤20% variation between troponin measurements
(iv) Must be taking aspirin prior to PCI
(v) Must be prescribed a second antiplatelet agent and statin therapy prior to PCI
(vi) PCI vessel caliber>2.5 mm diameter vessels
(vii) Obstructive coronary artery disease (defined as diameter stenosis >70%)
(vii) De novo lesion: defined by interventionalist
(viii) Patient and coronary lesion appropriate for Invasive Coronary Physiological Measurements, deemed by the interventional cardiologist at the time of index PCI

### 7.2. Exclusion Criteria

(i) Pregnant females or lactating females
(ii) Age younger than 18 years
(iii) Evidence of active infection/inflammatory conditions that might be associated with markedly elevated CRP levels or other inflammatory markers in the blood (e.g., active rheumatoid arthritis)
(iv) Taking anti-inflammatory therapies (e.g., corticosteroids)
  - Including colchicine
(v) Known hypersensitivity to colchicine
(vi) Noncompliance with medications
(vii) Patients not on or unable to take aspirin before or after PCI
(viii) Patients unable to take a second antiplatelet agent and/or statin therapy after PCI
(ix) Moderate renal impairment defined as creatinine clearance <45 ml/min
(x) Hepatic dysfunction defined as alanine aminotransferase 1.5 × upper limit of the normal range
(xi) Thrombocytopenia or leucopenia
(xii) Already on moderate-strong CYP3A4 inhibitors
(xiii) Severe left ventricular function defined as LVEF <35%
(xiv) Acute Myocardial Infarction in the last 12 months
(xv) Cardiogenic shock or hemodynamic instability
(xvi) ST-elevation myocardial infarction (STEMI)
(xvii) Patients who do not go on to PCI and/or patients who have PCI immediately prior to coronary artery bypass grafting
(xviii) Significant complex disease as deemed by interventionalist
  - Bifurcation lesions
  - Left main PCI or left main >50% stenosis
  - Chronic total occlusion of a vessel requiring PCI
  - Side branch involvement or occlusion
(xix) PCI to a small caliber vessel (<2.5 mm in diameter), distal vessel, or vessel supplying a small distal territory
(xx) Incomplete blood sampling
(xxi) PCI performed outside the allowed time frame of colchicine or placebo before treatment (6 to 24 hours prior to PCI)
(xxii) Unstable troponins or new ST elevation prior to PCI and after randomization
(xxiii) Unable to perform invasive coronary physiological measurements
(xxiv) Heavily calcified or tortuous vessels such that safe passage of the pressure wire could not be guaranteed
(xxv) Contraindications to adenosine

## Figures and Tables

**Table 1:** Baseline patient characteristics and pre-PCI medication use, by study drug randomization.

Baseline characteristics	Colchicine (*n* = 24)	Placebo (*n* = 26)
NSTEMI vs. SA presentation		
NSTEMI	11 (46)	12 (46)
SA	13 (54)	14 (54)
Baseline patient characteristics		
Age, *y*	67.0 ± 10.2	62.6 ± 11.7
Male	16 (67)	18 (69)
Obese (BMI >30)	12 (50)	19 (73)
Current smoker	4 (17)	6 (23)
Hypertension	13 (54)	15 (58)
Dyslipidaemia	15 (63)	20 (77)
Diabetes mellitus	8 (33)	5 (19)
Prior myocardial infarction	4 (17)	4 (15)
Prior percutaneous coronary intervention	3 (13)	4 (15)
Family history of ischaemic heart disease	11 (46)	18 (69)
Pre-PCI medication use		
Aspirin	24 (100)	26 (100)
P2Y_12_ inhibitor	23 (96)	25 (96)
Statins	23 (96)	22 (85)
Beta-blockers or/& Ca-channel blocker	16 (67)	22 (85)
Angiotensin-converting enzyme inhibitors	9 (38)	12 (46)
Angiotensin–II–receptor antagonists	5 (21)	7 (27)
Nitrates	2 (8)	2 (8)

Values are *n* (%) or mean ± SD.

**Table 2:** PCI characteristics, by study drug randomization.

PCI characteristics	Colchicine (*n* = 24)	Placebo (*n* = 26)	*p* value^*∗*^
Access			
Radial	20 (83)	21 (81)	1^||^
Femoral	4 (17)	5 (19)	1^||^
PCI artery			
Left anterior descending	13 (54)	12 (46)	0.57^§^
Intermediate	2 (8)	0 (0)	0.23^||^
Left circumflex	5 (21)	6 (23)	1^||^
Right coronary artery	4 (17)	8 (31)	0.33^||^
PCI procedure duration (minutes)	55 (47–69)	62 (51–70)	0.25^‡^
Contrast volume (mL)	150 (120–200)	160 (120–193)	0.86^‡^
Total number of balloon inflations	7.0 (5.0–11.0)	6.0 (5.0–9.0)	0.86^‡^
Total time of balloon inflations (seconds)	66 (48–80)	69 (51–91)	0.78^‡^
Total stent length (mm)	18 (15–24)	23 (18–32)	0.07^‡^
Maximum stent diameter (mm)	3.28 (2.95–3.99)	3.33 (3.00–3.64)	0.88^‡^
Stent type			
Drug-eluting stent	23 (96)	26 (100)	0.48^||^
Bare metal stent	1 (4)	0 (0)	0.48^||^
Pre-PCI balloon dilation	23 (96)	25 (96)	1^||^
Post-PCI balloon dilation	22 (92)	24 (92)	1^||^
Use of OCT or IVUS	0	0	
PCI complications			
Stent dissection	1/23 (4)	1 (4)	1^||^
No reflow	0	0	

Values are *n*/*N* (%), mean ± SD or median (LQ-UQ), stent dissection assessed by angiography, ^*∗*^denotes comparison between colchicine vs placebo group, † denotes independent-samples *t*-test, ‡ denotes Mann–Whitney-*U* test, § denotes Chi-squared test, and || denotes Fisher's exact test.

**Table 3:** Combined pre-PCI, post-PCI, and change in invasive coronary physiological and laboratory measurements, by study drug randomization.

	Colchicine (*n* = 24)	Placebo (*n* = 26)	*p* value^*∗*^
Invasive coronary physiological measurements			
FFR			
Pre-PCI	0.65 (0.47–0.79)	0.72 (0.52–0.80)	0.42^‡^
Post-PCI	0.88 (0.83–0.93)	0.90 (0.81–0.93)	0.79^‡^
Change	0.19 (0.09–0.38)	0.13 (0.07–0.33)	0.45^‡^
Tmn rest			
Pre-PCI	0.82 (0.57–1.55)	1.19 (0.54–1.59)	0.73^‡^
Post-PCI	0.89 (0.52–1.44)	0.64 (0.39–1.09)	0.16^‡^
**Change**	**0.01 (−0.30–0.23)**	**−0.31 (−0.80–0.01)**	**0.03** ^‡^
Tmn hyperaemia			
Pre-PCI	0.38 (0.31–0.69)	0.45 (0.20–0.78)	0.87^‡^
Post-PCI	0.26 (0.14–0.45)	0.26 (0.16–0.46)	0.57^‡^
Change	−0.12 (−0.43–0.11)	−0.12 (−0.43–0.04)	0.84^‡^
CFR			
Pre-PCI	2.10 (1.40–2.70)	1.95 (1.38–3.30)	0.92‡
**Post-PCI**	**3.25 (2.08–4.40)**	**2.00 (1.48–3.30)**	**0.03** ^**‡**^
**Change**	**1.20 (−0.30–2.10)**	**0.10 (−0.70–0.63)**	**0.03** ^**‡**^
BRI			
Pre-PCI	54.40 (21.83–96.63)	47.65 (26.63–106.00)	0.79^‡^
Post-PCI	73.55 (44.05–122.67)	54.37 (30.73–95.52)	0.25^‡^
Change	15.86 (−1.53–40.29)	−5.16 (−41.10–17.76)	0.052^‡^
IMR			
Pre-PCI	21.30 (11.50–26.80)	18.05 (9.95–32.28)	0.97^‡^
Post-PCI	17.02 (10.40–31.43)	19.92 (14.30–34.18)	0.27^‡^
Change	0.91 (−6.88–12.37)	4.90 (−11.34–11.66)	0.58^‡^
RRR			
Pre-PCI	3.26 (1.86–4.15)	2.75 (2.04–4.31)	0.94^‡^
**Post-PCI**	**4.25 (2.45–5.24)**	**2.75 (1.67–3.44)**	**<0.01** ^**‡**^
Change	1.05 (−0.91–2.64)	−0.09 (−1.34–0.88)	0.054^‡^
Laboratory results			
hs-troponin-I (ng/L)			
Pre-PCI	9 (3–769)	13 (3–93)	0.88^‡^
Post-PCI	184 (60–643)	259 (139–779)	0.55^‡^
**Absolute change**	**46 (1–154)**	**152 (48–633)**	**0.01** ^**‡**^
WBC count			
**Pre-PCI**	**6.7** ± **1.3**	**7.7** ± **1.5**	**0.02** ^**†**^
Post-PCI	7.6 ± 1.6	8.5 ± 1.7	0.06^†^
Change	0.9 ± 1.6	0.8 ± 1.0	0.84^†^
Neutrophil count			
Pre-PCI	4.0 ± 1.1	4.7 ± 1.3	0.06^†^
**Post-PCI**	**4.7** ± **1.1**	**5.6** ± **1.4**	**0.02** ^**†**^
Change	0.6 ± 1.3	0.9 ± 1.0	0.48^†^
hsCRP (mg/L)			
Pre-PCI	1.1 (0.5–5.7)	1.1 (0.6–4.3)	0.78^‡^
Post-PCI	1.0 (0.5–5.9)	1.7 (0.8–2.7)	0.97^‡^
Change	0.0 (−0.1–0.1)	0.2 (0.0–0.4)	0.21^‡^

Values are mean ± SD or median (LQ-UQ), ^*∗*^denotes comparison between colchicine vs placebo group, † denotes independent-samples *t*-test, and ‡ denotes Mann–Whitney *U* test.

**Table 4:** Primary study endpoint, by study drug randomization.

Primary endpoint	Colchicine (*n* = 24)	Placebo (*n* = 26)	*p* value^*∗*^
Combined CMVD: (CFR <2.0 or RRR <2.0 or IMR >32)	10 (42)	16 (62)	0.16^§^
RRR <2.0 or IMR >32	8 (33)	14 (54)	0.14^§^
CFR or RRR <2.0	5 (21)	12 (46)	0.06^§^
RRR <2.0	3 (13)	9 (35)	0.10^||^
CFR <2.0	5 (21)	12 (46)	0.06^§^
IMR >32	6 (25)	8 (31)	0.65^§^

Values are *n* (%), ^*∗*^denotes comparison between colchicine vs placebo group, § denotes Chi-squared test, and || denotes Fisher's exact test.

## Data Availability

Data were available on request.

## Abbreviations

ACS: Acute coronary syndrome
BMS: Bare metal stent
BRI: Baseline resistance index
CAD: Coronary artery disease
CFR: Coronary flow reserve
CRP: C-reactive protein
DES: Drug-eluting stent
FFR: Fractional flow reserve
hs: High-sensitive
IHD: Ischaemic heart disease
IL: Interleukin
IM: Intermediate
IMR: Index of microvascular resistance
IVUS: Intravascular ultrasound
LAD: Left anterior descending
LCx: Left circumflex
LQ-UQ: Lower quartile-upper quartile
NLRP3: Nucleotide-binding oligomerization domain-like receptor, pyrin domain-containing 3
NSTEMI: Non-ST-elevation myocardial infarction
OCT: Optical coherence tomography
PCI: Percutaneous coronary intervention
PM-Injury: Periprocedural myocardial injury
PPMI: Periprocedural myocardial infarction
RCA: Right coronary artery
RRR: Resistive Reserve Ratio
SA: Stable angina
SD: Standard deviation
Tmn_Rest_: Transit time under resting conditions
Tmn_Hyp_: Transit time at maximal hyperemia
URL: Upper reference limit
WBC: White blood cells.

## Additional Points

*Summary*. Impaired coronary microvascular function after percutaneous coronary intervention (PCI) is associated with worse outcomes. Anti-inflammatory medications may help. In this randomized placebo-controlled study, colchicine, a safe and widely available anti-inflammatory, given 6 to 24 hours before PCI improved postprocedure Resistive Reserve Ratio (RRR) and Coronary Flow Reserve (CFR). RRR is a newer marker representing the vasodilatory capacity of the coronary microcirculation and the cumulative functional disease burden throughout the interrogated vessel. It has superior prognostic value in stable and unstable coronary artery disease patients. In association with improved RRR and CFR, we found patients given preprocedural colchicine also had less PCI procedure-related troponin release and less inflammation.
